# Dihydronicotinamide Riboside Is a Potent NAD^+^ Precursor Promoting a Pro-Inflammatory Phenotype in Macrophages

**DOI:** 10.3389/fimmu.2022.840246

**Published:** 2022-02-25

**Authors:** Claudia C. S. Chini, Thais R. Peclat, Lilian S. Gomez, Julianna D. Zeidler, Gina M. Warner, Sonu Kashyap, Delaram Z. Mazdeh, Faisal Hayat, Marie E. Migaud, Aneel Paulus, Asher A. Chanan-Khan, Eduardo N. Chini

**Affiliations:** ^1^ Department of Anesthesiology and Perioperative Medicine, Mayo Clinic, Jacksonville, FL, United States; ^2^ Signal Transduction and Molecular Nutrition Laboratory, Kogod Aging Center, Department of Anesthesiology and Perioperative Medicine, Mayo Clinic College of Medicine, Rochester, MN, United States; ^3^ Mitchell Cancer Institute, University of South Alabama, Mobile, AL, United States; ^4^ Department of Pharmacology, College of Medicine, University of South Alabama, Mobile, AL, United States; ^5^ Division of Cancer Biology, Mayo Clinic, Jacksonville, FL, United States; ^6^ Division of Hematology and Oncology, Mayo Clinic, Jacksonville, FL, United States

**Keywords:** NAD, NRH, macrophages, inflammatory, gene expression

## Abstract

Nicotinamide adenine dinucleotide (NAD) metabolism plays an important role in the regulation of immune function. However, a complete picture of how NAD, its metabolites, precursors, and metabolizing enzymes work together in regulating immune function and inflammatory diseases is still not fully understood. Surprisingly, few studies have compared the effect of different forms of vitamin B3 on cellular functions. Therefore, we investigated the role of NAD boosting in the regulation of macrophage activation and function using different NAD precursors supplementation. We compared nicotinamide mononucleotide (NMN), nicotinamide riboside (NR), and nicotinamide (NAM) supplementation, with the recently described potent NAD precursor NRH. Our results show that only NRH supplementation strongly increased NAD^+^ levels in both bone marrow-derived and THP-1 macrophages. Importantly, NRH supplementation activated a pro-inflammatory phenotype in resting macrophages, inducing gene expression of several cytokines, chemokines, and enzymes. NRH also potentiated the effect of lipopolysaccharide (LPS) on macrophage activation and cytokine gene expression, suggesting that potent NAD^+^ precursors can promote inflammation in macrophages. The effect of NRH in NAD^+^ boosting and gene expression was blocked by inhibitors of adenosine kinase, equilibrative nucleoside transporters (ENT), and IκB kinase (IKK). Interestingly, the IKK inhibitor, BMS-345541, blocked the mRNA expression of several enzymes and transporters involved in the NAD boosting effect of NRH, indicating that IKK is also a regulator of NAD metabolism. In conclusion, NAD precursors such as NRH may be important tools to understand the role of NAD and NADH metabolism in the inflammatory process of other immune cells, and to reprogram immune cells to a pro-inflammatory phenotype, such as the M2 to M1 switch in macrophage reprogramming, in the cancer microenvironment.

## Introduction

The machinery that regulates extracellular and intracellular NAD levels and functions is complex, consisting of many degrading and synthesizing enzymes, as well as membrane channels and transporters. Several extracellular NAD-metabolizing enzymes rapidly degrade extracellular NAD^+^ and its precursors into metabolites such a nicotinamide mononucleotide (NMN), nicotinamide riboside (NR), adenosine diphosphate ribose (ADPR), adenosine, and nicotinamide (NAM) that can be further metabolized, serving as signaling molecules or precursors for NAD synthesis. Once taken up by cells, these NAD metabolites and vitamin B3 derivatives can be used directly or converted intracellularly into other metabolites that will be used to regenerate the intracellular NAD pool (1, 2).

Immune cells express many NAD synthesizing and degrading enzymes that are present intracellularly and extracellularly such as nicotinamide phosphoribosyltransferase (NAMPT)/nicotinate phosphoribosyltransferase (NAPRT), CD38, CD73, CD157, Poly (ADP-ribose) polymerases (PARPs) and sirtuins. The expression of these enzymes is regulated during inflammatory disease processes and appears to hold an important role in the function of different immune cell types (3–6). Functions such as pro-inflammatory response, immunosuppression, macrophage polarization, immune cell migration, DNA repair and epigenetics have been shown to be influenced by these enzymes and by alterations in NAD metabolism (7, 8), suggesting a role for the NAD metabolome in the regulation of immune function. However, a complete picture of how NAD, its metabolites, precursors, and metabolizing enzymes work together in regulating immune function and inflammatory diseases is lacking.

The role of NAD and its precursors in inflammatory responses of monocytes/macrophages has been controversial. Two pathways of NAD synthesis were shown to be important for macrophages to sustain their inflammatory phenotype. Production of NAD *via* the kynurenine pathway (KP), in the *de novo* pathway, was shown to be required to induce normal inflammatory macrophage response (9). Also, the NAD salvage pathway appears to be necessary for macrophages to satisfy their energy requirements and maintain their pro-inflammatory phenotype (10). In support of this hypothesis, the NAMPT inhibitor FK866 significantly downregulated the expression of M1 macrophage-specific genes, such as *Il1b, Il6, Tnf*, and *Nos2* in bone marrow-derived macrophages (BMDM) treated with IFN-γ and LPS or LPS alone (10). In the same study, NMN treatment upregulated the expression of M1-specific genes, suggesting that NMN may augment inflammation by promoting M1 macrophage polarization (10). In another study, NR-induced NAD^+^ boosting enhanced IL-1β release in LPS-primed human monocytes exposed to ATP *in vitro*, while the inflammatory responses in LPS-exposed monocytes were inhibited by NAD^+^ depletion with FK866 (11). In contrast, other studies provided evidence that NAD supplementation may suppress inflammation-induced cytokine production. NMN supplementation alleviated LPS-induced inflammation *via* decreasing secretion of the pro-inflammatory cytokines IL1 and IL6 in THP-1, Raw 264.7, and peritoneal macrophages, but did not affect basal release of these cytokines (12). Additionally, NR supplementation reverted NAD decline, induction of pro-inflammatory gene expression, and the increase in glycolytic capacity induced by ethanol in RAW 264.7 macrophages (13). These data together suggest that NAD and its precursors may have complex roles in macrophages during inflammatory processes.

Recently, two novel and potent NAD reduced precursors, NRH and NMNH, have been described and their roles been explored *in vitro* and *in vivo* (14–18). NRH and NMNH boost NAD^+^ levels faster, and to much higher levels than NR and NMN (14, 16–18). Also, the NAD^+^ boosting occurs independent of the enzymes NR kinase (NRK) and NAMPT (14), which are involved in NAD production in the salvage pathway. It appears that NMNH is converted to NRH outside of the cell, and NRH is the precursor that enters the cell to increase intracellular NAD^+^ levels (14, 18). Precursors such as NMN and NR can be degraded by extracellular enzymes (19–21), lowering their potential effects. It is believed that NRH is transported in its intact form across the cell membrane (2, 14), making it a powerful tool to understand the effects of NAD boosting in cellular processes. In addition, investigating the effects of these reduced NAD precursors can help us understand the physiological consequences of extreme boosting in NAD^+^ levels, and to develop therapies to metabolically reprogram immune cells. In addition, unlike other NAD precursors, NRH is converted to NADH first, before it is oxidized to NAD^+^ (14, 16). Therefore, in addition to boosting NAD^+^, NRH supplementation may overwhelm cells with NADH, which may have significant consequences to cell function. Our goal in the present study was to use NRH and other NAD precursors to investigate how NAD boosting regulates macrophage activation and function. Our results show that, different than supplementation with other NAD precursors, NRH supplementation can drive an inflammatory phenotype in resting macrophages through an adenosine kinase/ENT-IKK-dependent pathway. Precursors such as NRH might be important tools to reprogram immune cells to a pro-inflammatory phenotype.

## Material and Methods

### Reagents

The following reagents were used: NRH (14), NR (Laurus Labs), Olaparib (LC laboratories), 5-iodotubercidin (5-IT) (Selleckchem), nitrobenzylthioinosine (NBTI), ABT-702 (Cayman Chemical), adenosine 5’-(α,β-methylene) diphosphate (APCP) (Tocris), BMS 345541 (Selleckchem). NAM, NAD, NMN, ADH, Diaphorase, adenosine, phorbol myristate acetate (PMA), FK866, lypopolysaccharide (LPS) were obtained from Sigma-Aldrich.

### Animals

BMDM were isolated from 3-6 months C57BL/6 mice.

### Cell Culture

#### BMDM Isolation and Treatment

Mouse BMDM were isolated as previously described by Matalonga et al. (22). Briefly, BMDMs were obtained from bone marrow precursors differentiated for 6–7 days in DMEM supplemented with 20% heat-inactivated FBS and 30% L929 cell conditional media and 1% pen/strep solution (Gibco). Both female and male mice were used indistinctively for the generation of primary macrophages. BMDMs were seeded at 3 million cells per 35-mm dish and cultured in DMEM+ 2% FBS+ 1% pen/strep in the presence of LPS or NAD precursors for 6, 16, or 20 hours.

#### THP-1 Cells

THP-1 cells were obtained from ATCC and cultured in RPMI supplemented with 10% FBS and 1% pen/strep. To differentiate THP-1 from monocytes to macrophages, cells were treated with 50 ng/ml PMA for 48 hours. After 48 hours, media was changed to RPMI+ 1% FBS for 20 hours and then cells were incubated with NAD precursors for 6 hours.

#### Cell Viability Assay and Cell Counts

25,000 BMDM cells were plated into 96 well plates in DMEM+ 2% FBS+ 1% pen/strep in a volume of 180 μl. After 4-6 hours cells were treated with compounds. After 20-24 hours 20 μl of alamarBlue dye (Thermo Fisher) was added to wells and kept for 3 hours. Next, fluorescence was measured using a fluorescence excitation wavelength of 540–570 nm (peak excitation is 570 nm) and fluorescence emission at 580–610 nm (peak emission is 585 nm) in a SpectraMax Gemini XPS (Molecular Devices). 5 wells were used in each experiment for all experimental conditions. The experiment was repeated 4 times. When cell counts were determined we used the trypan blue (Gibco) exclusion test. Cell imaging was performed on EVOS XL Imaging System from Life Technologies.

### Immunoblotting

Immunoblottings for BMDMs were performed as previously described by Tarrago et al. (23). Cells were homogenized and lysed in NETN buffer (20 mM Tris-HCl (pH 8.0), 100 mM NaCl, 1 mM EDTA and 0.5% Nonidet P-40) supplemented with 50 mM β-glycerophosphate, 5 mM NaF and a protease inhibitor cocktail (Roche). For PARylation analysis 100 μM tannic acid was included in the lysis buffer. After 20 minutes of incubation on ice, the samples were centrifuged at 12,000 rpm for 10 minutes at 4°C. Protein concentrations in the supernatants were determined by Bio-Rad protein assay. Lysates were separated by SDS–PAGE, and electrophoretically transferred to polyvinylidene difluoride (PVDF) membranes (Immobilon-P; Millipore). Enhanced chemiluminescence detection was performed using SuperSignal West Pico or Femto Chemiluminescence Substrate (Thermo Scientific). Films were scanned and densitometry was performed using ImageJ. The following antibodies and their dilutions were used for immunoblotting: anti-mouse pp65 (S536) (Cell Signaling Technology; 3033, 1:1,000) anti-mouse p65 (Cell Signaling Technology; 8242, 1:1,000), actin (Cell Signaling Technology; 8457, 1:5,000), PAR (Trevigen, 4335, 1:1,000).

### Measurement of NAD^+^ Levels by Cycling Assay

Detection of NAD^+^ was performed using a in house cycling assay. The use of the cycling assay has potential limitations related with the sensitivity and the specificity of the measurements. To mitigate these limitations, we have previously extensively validated our assay for specificity and sensitivity. Our cycling assay is very specific for NAD^+^ and NADH and does not detect any of the other nucleotides that we have tested including NADP^+^, Nicotinamide, nicotinic acid, NAAD (nicotinic acid adenine dinucleotide), NAADP (nicotinic acid adenine dinucleotide phosphate), NGD (nicotinamide guanine dinucleotide), NHD (hypoxanthine dinucleotide), cADPR (cyclic-ADP-ribose), ATP, ADP, AMP, cAMP, and GTP. Importantly, we used an acid extraction that degrades NADH to only measure NAD^+^. Furthermore, in control experiments we have performed the cycling assay and correlated the results with side-by-side measurements with HPLC-MS. Extensive validation of the cycling assay has been previously described (20, 23, 24). In summary, to determine intracellular NAD^+^ levels, 3×10^6^ cells were homogenized in 10% trichloroacetic acid (TCA). Samples were centrifuged at 12,000 rpm for 2 minutes at 4°C. The supernatants were collected, and the pellets were resuspended in 0.2 N NaOH for protein determination. TCA was removed with organic solvents (three volumes of 1,1,2-trichloro-1,2,2-trifluroethane: one volume of trioctylamine) in a ratio of two volumes of organic solvent to one volume of sample. After phase separation, the top aqueous layer containing NAD^+^ was recovered and the pH was corrected by the addition of 1 M Tris (pH 8.0). For the cycling assay, samples were diluted in 100 mM sodium phosphate buffer (pH 8) in a volume of 100 μl per well and added to white 96-well plates. Next, 100 μl of reaction mix (0.76% ethanol, 4 μM flavin mononucleotide, 27.2U/ml alcohol dehydrogenase (ADH), 1.8 U/ml diaphorase and 8 μM resazurin) was added to each well. Then, 96-well plates were read in a fluorescence plate reader (Molecular Devices, SpectraMax Gemini XPS) in an excitation wavelength of 544 nm and an emission wavelength of 590 nm. We have previously demonstrated that the NAD^+^ cycling assay is as sensitive and specific as the ultra-performance liquid chromatography (UPLC)-mass spectroscopy (MS) assay.

### RNA Extraction and qPCR

Cells were lysed and RNA was isolated using Qiagen RNeasy kit. cDNA was synthesized using the ABI High Capacity cDNA Reverse Transcription kit. Real-time qPCR was performed using commercially available TaqMan gene expression probes (Applied Biosystems), according to the manufacturer’s instructions on a Bio-Rad CFX384 thermal cycler. The relative mRNA abundance of target genes was calculated by the 2^(−ΔΔCq)^ method. Expression changes were calculated relative to control cells or treatments. The Taqman probes used are described in **Tables 1** and **2**.

**Table 1:** Human Taqman probes.

Symbol	Gene Name	Assay #
*ACOD1*	Aconitate decarboxylase 1 (IRG1)	Hs00985781_m1
*ADK*	Adenosine Kinase	Hs00417073_m1
*BST1*	Bone marrow stromal cell antigen 1 (CD157)	Hs01070189_m1
*CCL2*	MCP1	Hs00234140_m1
*CD38*	CD38	Hs01120071_m1
*CXCL8*	IL-8	Hs00174103_m1
*GAPDH*	Glyceraldehyde phosphate dehydrogenase	Hs02758991_g1
*IL-1B*	Interleukin 1 beta	Hs01555410_m1
*IL-6*	Interleukin 6	Hs00985639_m1
*IL-10*	Interleukin 10	Hs00961622_m1
*NAMPT*	Nicotinamide phosphoribosyltransferase	Hs00237184_m1
*NMNAT1*	NMN adenylyltransferase 1	Hs00276702_m1
*NMNAT3*	NMN adenylyltransferase 3	Hs00736183_m1
*NOS2*	Nitric oxide synthase 2	Hs01075529_m1
*NT5E*	Ecto-5’-nucleotidase CD73	Hs00159686_m1
*PARP1*	Poly (ADP-Ribose) polymerase 1	Hs00242302_m1
*SARM1*	Sterile alpha and TIR motif containing 1	Hs00248344_m1
*SLC12A8*	NMN transporter	Hs00226405_m1
*SLC29A1*	ENT1 (Equilibrative nucleoside transporter 1)	Hs01085706_m1
*SLC29A2*	ENT2 (Equilibrative nucleoside transporter 2)	Hs01546959_g1
*TBP*	TATA box protein	Hs00427620_m1
*TNFA*	Tumor necrosis factor alpha	Hs01113624_g1

**Table 2:** Mouse Taqman probes.

Symbol	Gene Name	Assay #
*Adk*	Adenosine Kinase	Mm00612772_m1
*Bst1*	Bone marrow stromal cell antigen 1 (*Cd157*)	Mm00477672_m1
*Ccl2*	Monocyte chemotactic protein 1, Mcp1	Mm00441242_m1
*Ccl5*	Rantes	Mm01302427_m1
*Cd38*	CD38	Mm01220904_m1
*Cxcl1*	Gro-alpha	Mm04207460_m1
*Gapdh*	Glyceraldehyde 3-phosphate dehydrogenase	4352932E
*Il-1b*	Interleukin 1 beta	Mm00434228_m1
*Il-6*	Interleukin 6	Mm00446190_m1
*Il-12b*	Interleukin 12 beta	Mm01288989_m1
*Irg1*	Immune responsive gene 1	Mm01224532_m1
*Myc*	c-Myc	Mm00487804_m1
*Nampt*	Nicotinamide phosphoribosyltransferase	Mm00451938_m1
*Nmnat1*	NMN adenylyltransferase 1	Mm01257929_m1
*Nmnat3*	NMN adenylyltransferase 3	Mm00513791_m1
*Nos2*	iNOS	Mm00440502_m1
*Nt5e*	Ecto-5’- Nucleotidase Cd73	Mm00501910_m1
*Sarm1*	Sterile alpha and HEAT/Armadillo motif containing 1	Mm00555617_m1
*Slc12a8*	NMN transporter	Mm00506696_m1
*Slc29a1*	Ent1 (Equilibrative nucleoside transporter 1)	Mm01270577_m1
*Slc29a2*	Ent2 (Equilibrative nucleoside transporter 1)	Mm00432817_m1
*Tbp*	TATA box protein	Mm00446971_m1
*Tnfa*	Tumor necrosis factor alpha	Mm00443258_m1

### Mitochondrial Respiration

Mitochondrial oxygen consumption rates (OCR) were measured by high-resolution respirometry using an Oxygraph-2k instrument (Oroboros Instruments, Austria). 1-1.5 million BMDM cells were resuspended in 2ml of fresh DMEM 2% FBS and loaded into oxygraph chambers. Different respiratory parameters were assessed. Basal OCR was recorded after signal stabilization. Oligomycin addition (0.25 nM final concentration) allowed the evaluation of OCR uncoupled to ATP synthesis, referred here as leak respiration. The maximal OCR was measured in the presence of the optimum concentration of carbonyl cyanide p-(trifluoromethoxy) phenylhydrazone (FCCP), determined after FCCP titration. Data acquisition and analysis were carried out using DatLab6 software (Oroboros Instruments, Austria). Data were represented relative to the non-treated control for each cell preparation.

### Statistical Analysis

Data are mean ± SD analyzed by unpaired two-sided t-test or one-way ANOVA. Analyses were performed using Microsoft Excel 2010 and GraphPad Prism 9.2.0 software. Differences were considered statistically significant when P<0.05. *P < 0.05, **P < 0.01, *** P < 0.001, **** P < 0.0001.

## Results

### NRH Is a Potent NAD^+^ Precursor in BMDM

**Figure 1:**
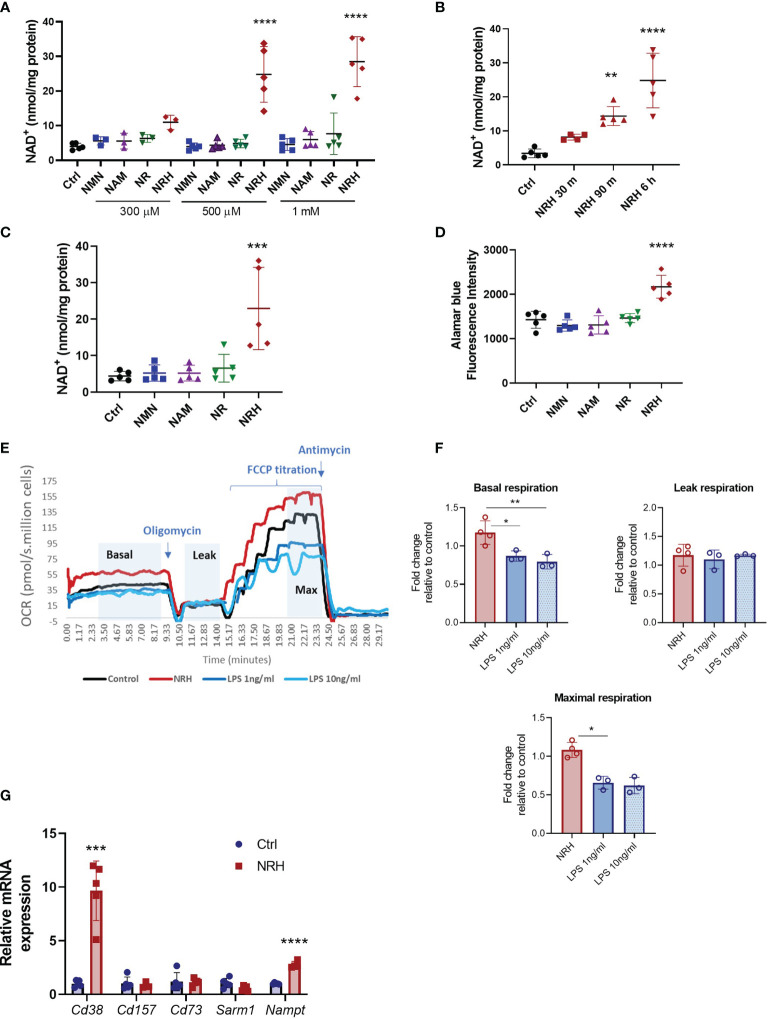
NRH is a potent NAD^+^ precursor in BMDM. **(A–C)** Intracellular NAD^+^ levels were measured after supplementation with NAD^+^ precursors (NMN, NAM, NR and NRH) or left untreated (Ctrl). **(A)** BMDM were supplemented with different doses of NAD^+^ precursors for 6 hours (n = 3-5). **(B)** BMDM were supplemented with 500 μM NRH for different times (n = 3-5). **(C)** BMDM were supplemented with 500 μM precursors for 16 hours (n = 5). **(D)** Cell viability was measured by alamarBlue reduction in BMDM treated for 20 hours with 500 μM precursors (n = 5). **(E, F)** Respirometry was measured in BMDM treated with 500 μM NRH or 1ng/ml and 10 ng/ml LPS for 20 hours. **(E)** Representative trace showing the oxygen consumption rate (OCR) during the experiment. **(F)** Fold-change in treated samples is expressed as relative to control untreated samples (n = 3-4). **(G)** mRNA levels measured in BMDM treated with 500 μM NRH for 6 hours. Expression in NRH-treated samples was calculated as fold change relative to the control untreated (n = 3-5). Data are mean ± SD. Significance was determined by comparing treatment groups with control untreated samples analyzed by one-way ANOVA with Dunnett’s multiple comparisons **(A–F)** or unpaired two-sided t-test **(G)**. *P < 0.05, **P < 0.01, ***P < 0.001, ****P < 0.0001.

Recently, NRH has been characterized as a potent NAD^+^ precursor in cell lines such as AML-12, NIH 3T3, 293T and HepG3 (14, 16). However, its effect on immune cells has not been described. In BMDM treatment with NRH for 6 hours induced a dose-dependent increase in NAD^+^ to levels 6-7 times higher than control non-treated cells (**Figure 1A**). In contrast, 6 hours supplementation with NMN, NR or NAM did not produce significant increases in NAD^+^ levels in BMDM for most of the concentrations tested (**Figure 1A**). We also observed that 500 μM NRH induced a rapid increase in intracellular NAD^+^ levels in BMDM, with a small increase detected as soon as 30 min (**Figure 1B**). Increases in NAD^+^ levels induced by NRH were similar at 6 and 16 hours of treatment, indicating that the increase is sustained for many hours (**Figures 1B, C**). Other NAD^+^ precursors did not induce NAD^+^ boosting in BMDM after 16 hours supplementation (**Figure 1C**). Moreover, we found that NRH also increased NAD^+^ levels in B (Ramos, JVM-13) and T (Jurkat) lymphoblasts (**Supplementary Figures 1A–C**). In B and T cells we found that NR could also increase NAD^+^ levels significantly. However, in all cell types tested, NRH was a more potent NAD^+^ precursor (**Supplementary Figures 1A–C**).

We then investigated the functional consequences of this large increase in NAD^+^ levels in BMDM. To determine the impact of NRH supplementation on parameters such as cell viability and oxygen consumption we performed a longer treatment with NRH (20 hours). Supplementation with 500 μM NRH increased BMDM cell viability, as determined by staining with alamarBlue, unlike other NAD^+^ precursors (**Figure 1D**). This increase in cell viability indicates that in BMDM NRH is not showing cytotoxic effects during our treatment period but, on the contrary, it appears to be improving cell survival. To confirm these results, we also determined cell viability and survival by cell counting and cellular visualization after 20 hours of supplementation with 500 μM NRH (**Supplementary Figures 1D, E**), or lipopolysaccharide (LPS) treatment (**Supplementary Figure 1D**). We did not observe a deleterious effect of NRH or LPS on the cell viability during the treatment period (**Supplementary Figures 1D, E**). We also investigated whether NRH supplementation altered oxygen consumption rates (OCR). Treatment of BMDM with 500 μM NRH for 20 hours did not significantly change the basal, leak, or maximum respiration in comparison to non-stimulated cells (**Figures 1E, F** and **Supplementary Figure 1F**). As a control, we treated BMDM with 1 or 10 ng/ml LPS and found that both concentrations decreased maximum respiration, differently than NRH (**Figure 1F**).

Next, we determined whether NRH treatment modified the expression of NAD^+^ synthesizing and degrading enzymes expressed by macrophages. To investigate the NRH effect on gene expression we treated BMDM with NRH for 6 hours, a time where NAD^+^ levels reach maximum increase in these cells. Treatment of BMDM with NRH for 6 hours increased mRNA expression of *Cd38* (NADase) and *Nampt* (NAD synthesizing enzyme) (**Figure 1G**), likely promoting an increase in the turnover of NAD metabolism. However, since NRH bypasses both CD38 and NAMPT, the consequences of these enzymes to NRH metabolism are likely small. These two enzymes are known to be induced by agents that promote a pro-inflammatory (M1-like) phenotype in macrophages, like LPS (20). In view of these observations, we next investigated the role of NRH in macrophage activation and inflammatory responses.

### NRH Supplementation Promotes the Expression of Pro-Inflammatory Genes in BMDM

**Figure 2:**
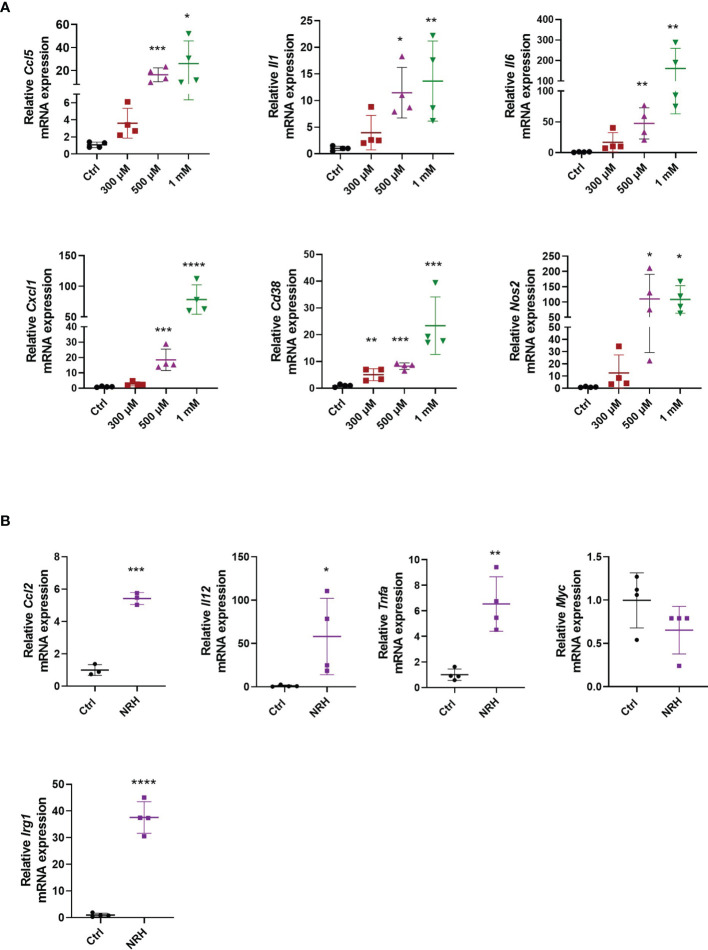
NRH promotes a pro-inflammatory phenotype in BMDM. **(A)** BMDM were supplemented with different doses of NRH for 6 hours. mRNA levels were measured and expressed as relative to the control untreated sample (Ctrl) (n = 3-4). **(B)** BMDM were supplemented with 500 μM NRH for 6 hours. mRNA levels were measured and expressed as relative to the control untreated sample (Ctrl) (n = 3-4). Data are mean ± SD. Significance was determined by comparing treatment groups with control untreated samples analyzed by one-way ANOVA with Dunnett’s multiple comparisons **(A)** or unpaired two-sided t-test **(B)**. *P < 0.05, **P < 0.01, ***P < 0.001, ****P < 0.0001.

To determine whether NRH regulates activation markers in macrophages, we treated BMDM with increasing doses of NRH (300–1000 μM) for 6 hours, and then assessed gene expression of several cytokines, chemokines, and enzymes by RT-qPCR. NRH treatment induced a marked dose-dependent increase in the mRNA expression of genes that are markers of a macrophage M1 phenotype, such as *Ccl5, Il1, Il6, Cxcl1* (**Figure 2A**) (25). In addition, the expression of *Cd38* and *Nos2*, two enzymes that are also markers of M1 macrophage phenotype (25), were significantly increased by most of the NRH concentrations tested (**Figure 2A**). We also examined the effect of 500 μM NRH on the expression of other genes, such as *Ccl2, Il12*, and *Tnfa*, and found that NRH caused at least a fivefold increase in the expression level of these cytokines/chemokines (**Figure 2B**). In contrast, *Myc* expression, a marker of the macrophage M2 phenotype (26), was not increased after NRH treatment, showing a trend for decreased expression (**Figure 2B**). We also examined *Irg1* expression in macrophages supplemented with NRH. Irg1 (ACOD1) is an enzyme involved in itaconate production and is induced by agents that promote inflammation in macrophages, like LPS (27, 28). Consistent with other markers of inflammation, *Irg1* expression was also markedly increased by NRH supplementation (**Figure 2B**).

**Figure 3:**
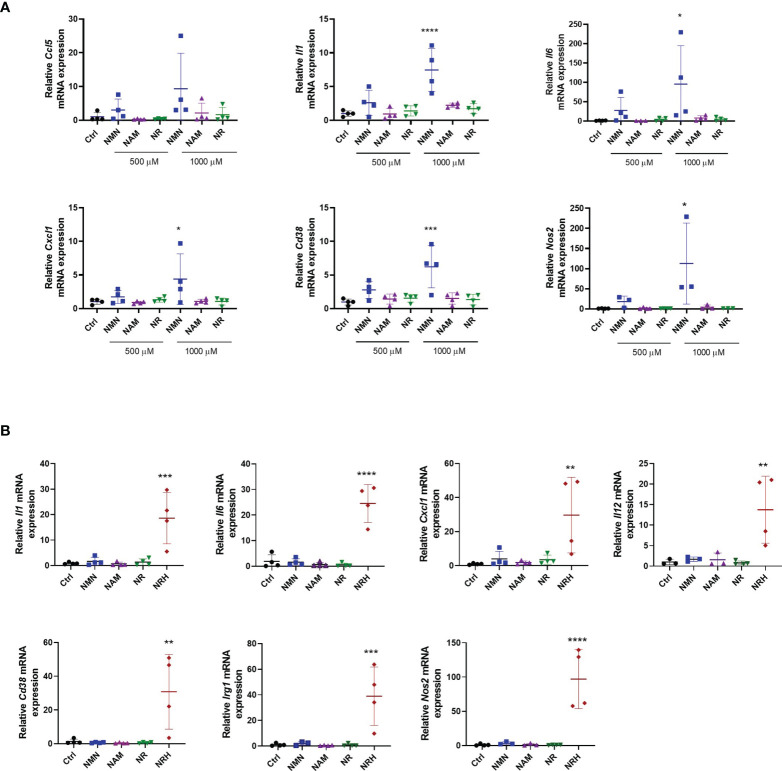
Other NAD^+^ precursors do not promote a pro-inflammatory phenotype to the same extent as NRH in BMDM. **(A)** BMDM were supplemented with different doses of precursors for 6 hours. mRNA levels were measured and expressed as relative to control untreated sample (Ctrl) (n = 3-4). **(B)** BMDM were supplemented with 500 μM precursors for 16 hours. mRNA levels were measured and expressed as relative to the control untreated sample (Ctrl) (n = 3-4). Data are mean ± SD. Significance was determined by comparing treatment groups with control untreated samples analyzed by one-way ANOVA with Dunnett’s multiple comparisons. *P < 0.05, **P < 0.01, ***P < 0.001, ****P < 0.0001.

We next investigated whether treatment with other NAD precursors at 500-1000 μM for 6 hours induced similar effects on gene expression as observed with NRH supplementation. Of all the precursors tested only NMN supplementation increased the expression of some cytokines/chemokines, *Cd38* and *Nos2*. This NMN effect was noted especially at 1000 μM, but it was smaller and less consistent compared to NRH (**Figure 3A**). Although no significant increase in NAD^+^ levels were detected by NMN supplementation at the time point tested (**Figures 1A, C**), we cannot exclude the possibility that NMN transiently increases NAD^+^ and regulates gene expression through this mechanism.

When gene expression of activation markers was measured 16 hours after treatment with NAD^+^ precursors, we observed that only NRH supplementation was able to sustain an effect on gene expression. The expression of several cytokines/chemokines, *Cd38, Nos2*, and *Irg1* were still increased after 16 hours of treatment with NRH (**Figure 3B**). This prolonged effect on gene expression is consistent with the NRH effect on NAD^+^ levels (**Figure 1C**), which is still increased after 16 hours.

### NRH Regulates Cytokine Production in THP-1 Cells

**Figure 4:**
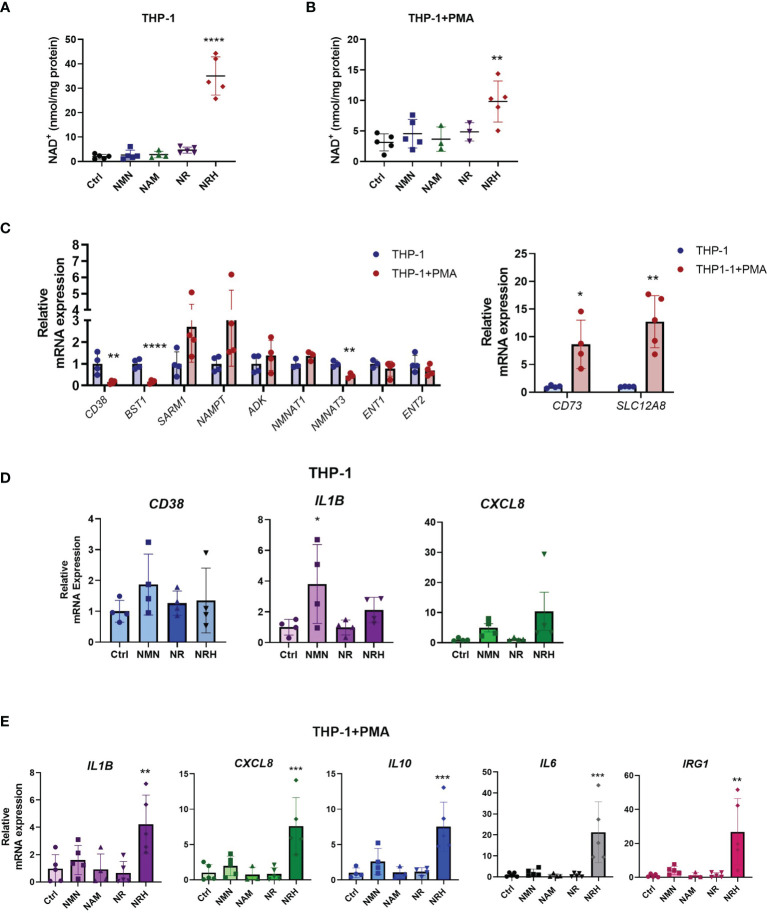
NRH regulates pro-inflammatory gene expression in THP-1 cells. **(A, B)** THP-1 cells were treated for 6 hours with 500 μM NAD precursors and intracellular NAD^+^ levels were measured (n = 3-5). **(A)** Undifferentiated THP-1 were used (monocytes). **(B)** PMA-treated THP1 were used (macrophage-like). **(C)** mRNA expression in PMA-treated THP-1 cells is shown as relative to expression in non-treated THP-1 cells (n = 3-5). **(D)** THP-1 cells were supplemented with 500 μM NAD precursors for 6 hours. mRNA expression was measured by qPCR. Expression is relative to the control untreated sample (Ctrl) (n = 4). **(E)** THP-1+PMA cells were supplemented with 500 μM NAD precursors for 6 hours. mRNA expression was measured by qPCR. Expression is relative to the control untreated sample (Ctrl) (n = 3-5). Data are mean ± SD. **(A, B, D, E)** Significance was determined by comparing treatment groups with control untreated samples analyzed by one-way ANOVA with Dunnett’s multiple comparisons. **(C)** Significance was determined by comparing mRNA expression in PMA-treated THP-1 cells with non-treated THP-1 cells analyzed by unpaired two-sided t-test for each gene. *P < 0.05, **P < 0.01, ***P < 0.001, ****P < 0.0001.

To further confirm the role of NRH on NAD^+^ boosting and regulation of gene expression in immune cells, we investigated the effect of NRH in THP-1 cells, a human monocytic leukemia cell line. This cell line can be differentiated into a macrophage-like phenotype using agents like phorbol 12-myristate 13-acetate (PMA) (29). When THP-1 cells were supplemented with NAD^+^ precursors, we found that only NRH was able to significantly increase NAD^+^ levels (**Figure 4A**). In THP-1 cells treated with PMA, NRH supplementation also caused a significant increase in NAD^+^ levels, but this increase was smaller compared to non-PMA-treated THP-1 cells (**Figure 4B**).

To investigate the reason behind the differences observed in non-treated and PMA-treated THP-1 cells, we measured the mRNA levels of NAD metabolism enzymes under these two conditions. When PMA-treated THP-1 cells were compared to non-treated cells, there was a significant decrease in mRNA expression of enzymes such as *CD38, CD157*, and *NMNAT3*. In contrast, gene expression of *CD73* and the putative NMN transporter *SLC12A8* (30) were increased, while other enzymes and transporters did not significantly change (**Figure 4C**). Since there were several changes in the pattern of gene expression for NAD metabolism enzymes between THP-1 untreated and PMA-treated cells, some of those changes may be contributing to the lower NAD^+^ boosting effect of NRH in PMA-treated THP-1 cells.

When cytokine gene expression was measured in non-treated THP-1 cells, NRH did not show a significant effect on increasing gene expression (**Figure 4D**), even though it promoted a large increase in NAD^+^ levels in these cells (**Figure 4A**). However, there was a trend for both NRH and NMN, but not NR, to increase pro-inflammatory gene expression. THP-1 cells express the enzymes and transporters necessary for NRH-induced NAD boosting, in addition to CD38 (which degrades NMN), CD157/BST1 (which degrades NR), and the putative NMN transporter. Since CD157/BST1 appears to be abundant in THP-1 cells (31), this could be a possible reason why NR is less effective than NMN in THP-1 cells.

In contrast, PMA-treated THP-1 cells, show an increase in expression levels of several cytokines upon NRH supplementation, as seen in BMDM (**Figure 4E**). Supplementation with other NAD^+^ precursors did not significantly increase gene expression. These different patterns of responses observed in macrophages and monocytes indicate that the NAD metabolome and the machinery to regulate inflammatory responses varies between immune cell types, highlighting the importance of characterizing the role of these precursors in several cell types.

### NRH Regulates the Expression of Pro-Inflammatory Genes in BMDM Through an ENT-ADK-Dependent Pathway

We next set out to determine whether the effect of NRH in inducing a pro-inflammatory phenotype in macrophages was related to its effect on NAD^+^ boosting. For that reason, we investigated whether the transporters and enzymes that have been shown to regulate entry and conversion of NRH into NAD^+^ in cells also regulate the NRH effect on gene expression.

**Figure 5:**
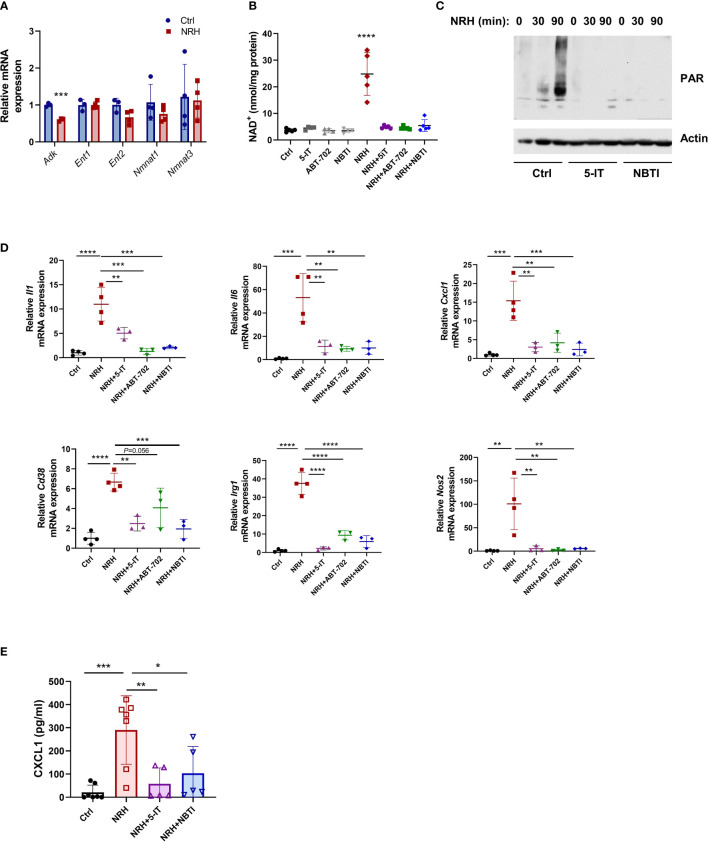
NRH regulates pro-inflammatory gene expression in BMDM through an ENT-ADK-dependent pathway. **(A)** BMDM were supplemented for 6 hours with 500 μM NRH or left untreated. mRNA expression of NRH-treated samples was calculated as fold change relative to the control untreated sample (n=3-4). **(B–D)** BMDM were pretreated for 1.5 hours with the following inhibitors: 1 μM 5-IT, 10 μM ABT-702, and 10 μM NBTI. **(B)** After inhibitor treatment, cells were supplemented or not with 500 μM NRH for 6 hours. Then, cells were collected and NAD^+^ levels were measured (n=4-5 biologically independent samples). **(C)** After inhibitor treatment, cells were supplemented or not with 500 μM NRH for 30 or 90 minutes. Figure shows a representative immunoblot where protein lysates were blotted with anti-PAR and anti-actin antibodies. **(D)** After inhibitor treatment, cells were supplemented or not with 500 μM NRH for 6 hours. mRNA expression was measured by qPCR and expressed as relative to the control untreated sample (Ctrl) (n = 3-4). **(E)** CXCL1 protein levels in the cell culture media were measured by ELISA (n = 5-7). Data are mean ± SD. In A and B significance was determined by comparing treatment groups with control untreated samples analyzed by one-way ANOVA with Dunnett’s multiple comparisons test. In **(D, E)** significance was determined by comparing all treatment groups analyzed by one-way ANOVA with Tukey’s multiple comparisons test. *P < 0.05, **P < 0.01, ***P < 0.001, ****P < 0.0001.

NRH, like NR, appears to be transported into the cell mainly by equilibrative nucleoside transporters (ENTs) (14, 32). Once inside the cell, NRH appears to be phosphorylated by adenosine kinase (ADK) into NMNH and subsequently converted to NADH by NMNATs (14, 17, 33). We first confirmed whether these enzymes were expressed in resting and NRH-treated macrophages. mRNA expression of *Adk, Ent1, Ent2, Nmnat1* and *Nmnat3* was observed in resting BMDM and supplementation with 500 μM NRH for 6 hours did not significantly change expression of most of these genes in BMDM. The only exception was *Adk*, which showed decreased, but still detectable, expression (**Figure 5A**).

The effect of NRH in NAD^+^ boosting has been shown to be blocked by the ENT inhibitor S-(4-nitrobenzyl)-6-thioinosine (NBTI) and the ADK inhibitors 5-iodotubercidin (5-IT) and ABT-702 in other cells (14). Pre-treatment of BMDM with all three inhibitors before NRH supplementation for 6 hours completely prevented the NAD^+^ boosting induced by NRH, while they did not affect NAD^+^ levels in control untreated cells (**Figure 5B**). NRH supplementation also induced a marked increase in poly-ADP-ribosylation (PAR), which is a measurement of PARP activity. This effect was blocked by 5-IT and NBTI, indicating that these inhibitors prevent the NRH-induced NAD*^+^* boosting and activation of downstream effectors such as PARP (**Figure 5C**).

By measuring mRNA expression, we found that 5-IT, ABT-702, and NBTI blocked the NRH-induced increase in the expression of *Il1, Il6, Cxcl1, Cd38, Irg1* and *Nos2*, but had mostly no effect in the absence of NRH (**Figure 5D** and **Supplementary Figure 2A**). Importantly, we measured the levels of the chemokine CXCL1 in the cell culture media of cells treated with NRH for 6 hours (**Figure 5E**) and 16 hours (**Supplementary Figure 2B**). Supplementation with NRH increased protein levels of CXCL1 in the media (**Figure 5E** and **Supplementary Figure 2B**), and this effect was blocked by pre-treatment with the inhibitors 5-IT and NBTI (**Figure 5E**). The CXCL1 levels were higher at 16 hours than at 6 hours, indicating a time-dependent accumulation of this chemokine in the media. These results together indicate that the NRH effect on NAD^+^ levels and pro-inflammatory responses in macrophages occurs through an ENT-ADK-dependent pathway.

To further explore the mechanisms by which NRH regulates macrophage cytokine/chemokine expression we pretreated BMDM with compounds that inhibit other enzymes involved in NAD metabolism. We wanted to determine whether these compounds regulate NAD^+^ levels or prevent pro-inflammatory gene expression when added before supplementation with NRH. In resting BMDM addition of the NAMPT inhibitor FK866 decreased NAD^+^ levels, but it did not affect the NAD^+^ boosting induced by NRH (**Supplementary Figure 2C**). Inhibition of PARP (with Olaparib), CD73 (with APCP), or addition of the immunosuppressive drug adenosine had also no effect on both basal and NRH-dependent NAD^+^ boosting (**Supplementary Figure 2C**). Except for the immunosuppressive effect of adenosine on NRH-induced *Il6* expression, and the effect of FK866 on *Il6* expression, the above compounds did not significantly alter the NRH-induced regulation of gene expression (**Supplementary Figure 2D**). These results indicate that the effect of NRH on NAD^+^ boosting and cytokine gene expression occurs independently of NAMPT, PARP, or CD73.

### NRH Supplementation in Combination With LPS Further Increased the Pro-Inflammatory Phenotype

**Figure 6:**
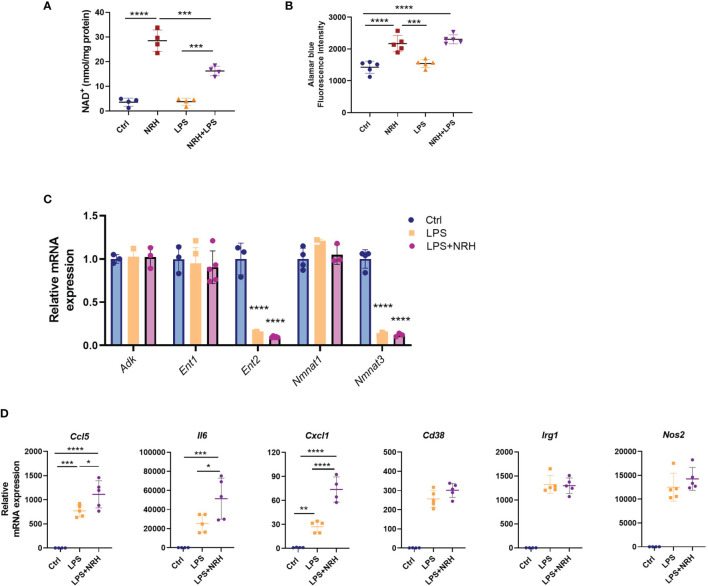
NRH supplementation in combination with LPS. **(A, B)** BMDM were supplemented with 500 μM NRH, 1 ng/ml LPS, combination of NRH and LPS (NRH+LPS) or left untreated (Ctrl). **(A)** NAD^+^ levels were measured after 6 hours treatments (n = 4). **(B)** Cell viability was measured by alamarBlue after 20 hours (n = 5). **(C)** mRNA expression of LPS and LPS+NRH-treated samples were calculated as relative to the control untreated (n = 3-5). **(D)** mRNA expression of LPS and LPS+NRH-treated samples were calculated as relative to the control untreated samples (n = 5). Data are mean ± SD. Significance was determined by comparing treatment groups analyzed by one-way ANOVA with Tukey’s multiple comparisons test **(A, B)** or Dunnett’s multiple comparisons test **(C, D)**. *P < 0.05, **P < 0.01, ***P < 0.001, ****P < 0.0001.

The findings described above demonstrate that NRH supplementation can produce a pro-inflammatory phenotype in resting macrophages. Therefore, next we wanted to explore the effects of NRH in combination with inflammatory stimuli such as LPS. When BMDM were treated with NRH alone for 6 hours, NRH promoted a nearly 8-fold increase in NAD^+^ levels. However, when a low concentration of LPS (1 ng/ml) was added 1 hour after NRH addition, NRH promoted only a fourfold increase in NAD^+^ levels, even though LPS alone at this concentration and time did not change NAD^+^ levels (**Figure 6A**). This difference was not due to a decrease in cell viability when LPS was added together with NRH, since the viability of NRH or NRH+LPS-treated macrophages for 20 hours was similar and higher than non-stimulated cells (**Figure 6B**). Also, LPS treatment alone did not decrease cell viability (**Figure 6B** and **Supplementary Figure 1D**).

It is known that LPS treatment promotes changes in the expression of many enzymes involved in NAD metabolism (20, 34, 35). Thus, we next investigated the effect of LPS on the mRNA expression of enzymes/transporters involved in NRH-induced NAD^+^ boosting. Interestingly, while expression of *Adk, Ent1*, and *Nmnat1* did not change after 6 hours of treatment with LPS, there was a major decrease in expression of *Ent2* and *Nmnat3* (**Figure 6C**). The same pattern of expression was observed when LPS was added in combination with NRH (**Figure 6C**). These results suggest that LPS may be inhibiting the expression of enzymes that are important for the NRH-induced NAD^+^ boosting effect.

LPS is a potent inducer of the macrophage M1 phenotype, even at lower concentrations. 6 hours treatment of BMDM with 1 ng/ml LPS strongly induced expression of many cytokines such as *Ccl5*, *Cxcl1* and *Il6*, and the enzymes *Cd38, Nos2* and *Irg1* in comparison to untreated control cells (**Figure 6D**). When NRH and LPS were added together, there was no additive effect on the increase in expression of *Cd38, Nos2* and *Irg1* compared to LPS alone. However, the combination of NRH+LPS treatment increased the expression of cytokines/chemokines to higher levels than LPS alone (**Figure 6D**), suggesting that NRH-induced NAD^+^ boosting can potentiate the effect of inflammatory stimuli on cytokine gene expression. The decrease in NAD^+^ levels promoted by high doses of LPS (100 ng/ml) (9, 10) may represent a negative feedback mechanism for its cytokine-producing effect that, as demonstrated here, can be at least partially bypassed by boosting NAD with NRH.

### NRH-Induced NAD-Boosting and Pro-Inflammatory Gene Expression Is Dependent on IKK

**Figure 7:**
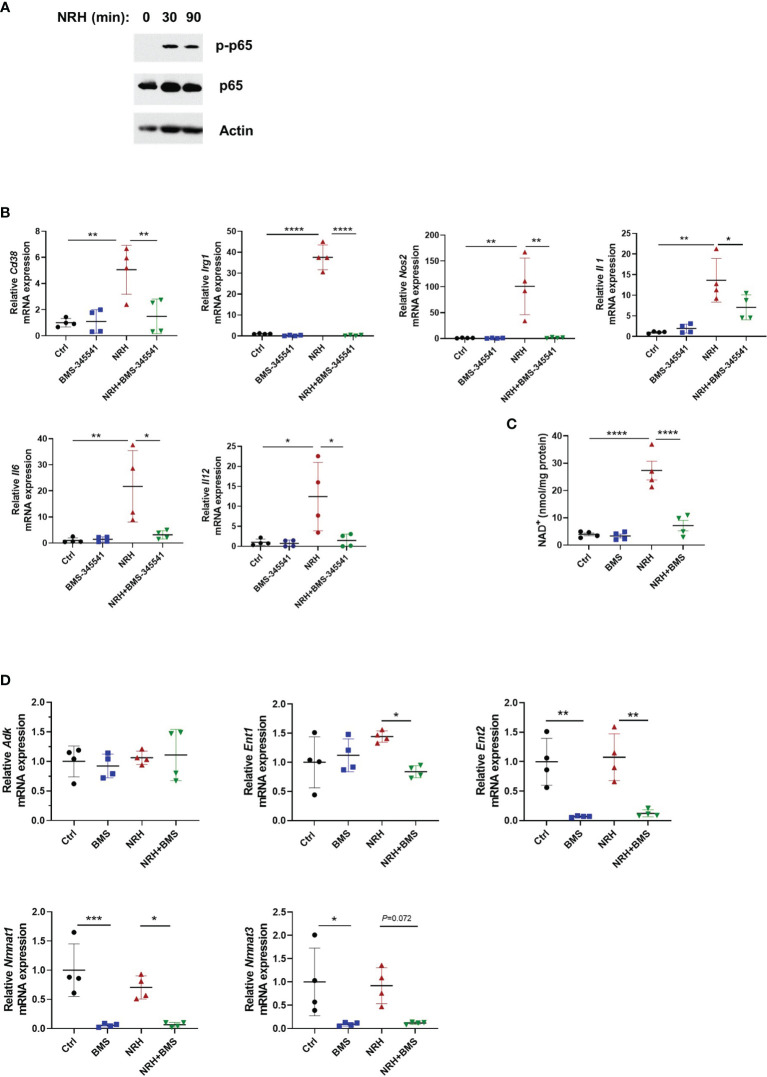
NRH-induced pro-inflammatory gene expression is dependent on the IKK/NF-κB pathway. **(A)** BMDM were supplemented with 500 μM NRH for 30 or 90 minutes or left untreated. Figure shows a representative immunoblot where protein lysates were blotted with anti-p-p65 (S536), anti-p65, and anti-actin antibodies. **(B)** BMDM were pretreated for 1.5 hours with 5 μM BMS-345541, an IKK inhibitor, before 500 μM NRH supplementation for 6 hours. mRNA expression was measured by qPCR and was expressed as relative to the control untreated samples (Ctrl) (n=4). **(C)** BMDM were pretreated for 1.5 hours with 5 μM BMS-345541. Then, cells were left untreated or supplemented with 500 μM NRH for 6 hours before cells were collected for NAD^+^ level measurements (n = 4). **(D)** BMDM were pretreated or not for 1.5 hours with 5 μM BMS-345541. Then, cells were left untreated or supplemented with 500 μM NRH for 6 hours. mRNA expression was measured by qPCR and was expressed as relative to the control untreated samples (Ctrl) (n = 4). Data are mean ± SD. Significance was determined by comparing all treatment groups analyzed by one-way ANOVA with Tukey’s multiple comparisons test. *P < 0.05, **P < 0.01, ***P < 0.001, ****P < 0.0001.

An important pathway that regulates gene expression of various pro-inflammatory genes including cytokines and chemokines in immune cells is the NF-κB pathway (36). To better understand the mechanism by which NRH regulates cytokine/chemokine gene expression we investigated if supplementation with NRH activates the NF-κB pathway in BMDM. Phosphorylation plays an important role in the activation of several components of the NF-κB pathway. The increase in phosphorylation of the subunit p65 at S536 has been used as a marker of NF-kB activation. Treatment of BMDM for 30 and 90 minutes with NRH caused a small increase in p65 levels and a marked increase in phosphorylation of S536 on NF-κB p65 subunit, suggesting that NRH supplementation is activating the NF-κB pathway (**Figure 7A**).

These results prompted us to test whether the NF-κB pathway was involved in the NRH effect on BMDM. The canonical pathway for NF-κB activation involves the multi-subunit IκB kinase (IKK) complex, which when activated by different stimuli phosphorylates IκBα, an NF-κB inhibitor. Phosphorylation of IκBα triggers ubiquitin-dependent IκBα degradation, releasing NF-κB to translocate to nuclei and activate gene expression (36). As shown in **Figure 7B**, pre-treatment of BMDM with 5 μM BMS-345541, a highly selective inhibitor of the catalytic subunit of IKK (37), blocked the effect of NRH on pro-inflammatory genes, suggesting a role for IKK on this pathway. Since the effect of NRH on gene expression in BMDM appears to be dependent on its NAD^+^-boosting effect (**Figure 5**), we then investigated if pre-treatment with BMS-345541 interferes with the NAD^+^-boosting effect of NRH. Surprisingly, IKK inhibition with BMS-345541 blocked the NAD^+^-boosting induced by NRH, suggesting that IKK may regulate components of the NRH pathway (**Figure 7C**).

**Figure 8:**
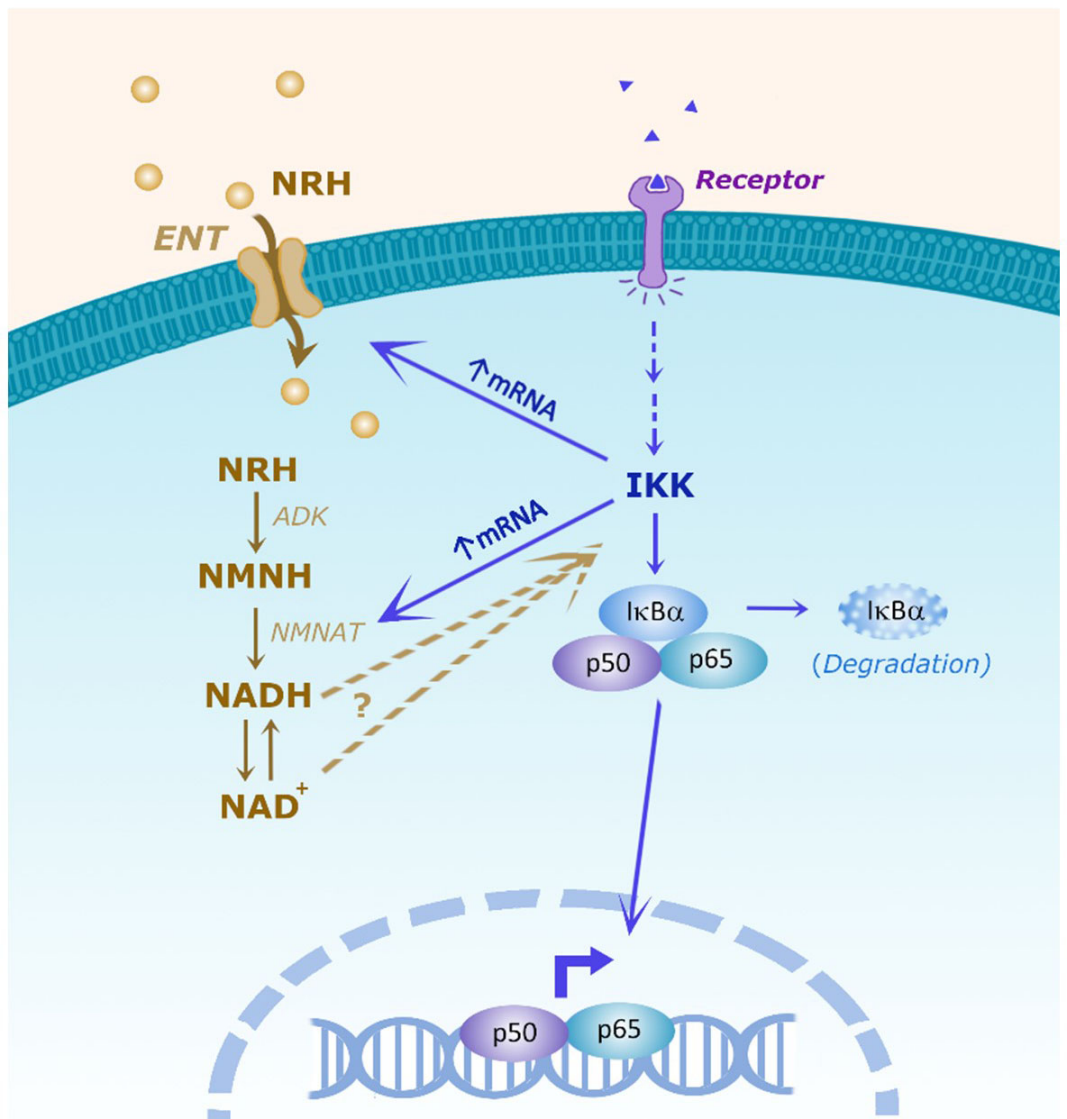
Scheme of the hypothesis showing the interaction between the NRH and NF-kB pathways. It has been proposed that NRH enters the cell through equilibrative nucleoside transporters (ENTs). Inside the cell NRH is phosphorylated by adenosine kinase (ADK) into NMNH. Then, NMNATs catalyze the transition from NMNH to NADH, which will then be converted to NAD^+.^ In the NF-kB pathway, membrane receptor activation promotes intracellular IKK activation which can have NF-kB-dependent and independent effects. In the NF-kB pathway, IKK can phosphorylate the inhibitor IkBα, targeting IkBα for degradation, releasing p50/p65 to regulate cytokine gene expression. NRH, through NAD boosting, activate the NF-kB pathway and pro-inflammatory gene expression, but the molecular mechanism involved remains to be determined. Conversely, IKK regulates the NRH-induced NAD^+^ boosting and mRNA expression of ENTs and NMNATs, indicating the existence of multiple connections between these two pathways.

To understand why IKK inhibition regulated the NRH-induced NAD^+^ boosting we examined the expression of enzymes involved in NRH-induced NAD^+^ boosting. Interestingly, BMS-345541 pre-treatment decreased the expression of *Nmnat1, Nmnat3*, and *Ent2* by 90% and the expression of *Ent1* by 40% in the presence of NRH (**Figure 7D**). All together, these results suggest that in BMDM NRH activates the NF-κB pathway and pro-inflammatory gene expression, and that the effects of NRH in NAD^+^ boosting and gene expression are regulated by IKK (**Figure 8**).

## Discussion

It is becoming clear that metabolite availability promotes changes in cell function and fate of immune cells (38). Macrophages, for example, can undergo profound metabolic reprogramming in response to metabolite availability (5, 38). In addition, many metabolic changes occur in activated macrophages to support and regulate the gene expression of inflammatory mediators (5, 8, 38). Previous studies have investigated the role of NAD metabolism during macrophage activation induced by LPS. However, these studies offer conflicting results. While studies show that the salvage pathway is required for macrophages to maintain an inflammatory response (10), others report that NAD^+^ boosting in activated macrophages may decrease the pro-inflammatory phenotype (12, 13). Since these studies were performed in different types of macrophages and used different NAD precursors and experimental conditions, a clear picture of the role of NAD in macrophage responses is still missing.

To better understand the role of NAD metabolism during pro-inflammatory macrophage activation, we investigated the effect of supplementation with different NAD^+^ precursors on macrophage activation. The NAD^+^ precursors NMN and NR have been extensively studied and supplementation with these NAD^+^ intermediates has shown preventive and therapeutic effects, ameliorating age-associated pathophysiology and disease conditions (2, 39, 40). To determine whether NAD precursors regulate the inflammatory phenotype in macrophages, we performed our supplementation experiments in resting BMDM, comparing the effects of NMN, NR, and NAM with the recently described precursor NRH. Our results together show that, of all the NAD precursors tested, NRH was the only one capable of strongly increasing NAD^+^ levels in macrophages. Importantly, under NRH supplementation BMDM and THP-1 macrophages became pro-inflammatory, showing that NAD boosting can drive inflammation in resting macrophages.

Previous studies in other cell culture types and mice have also shown that NRH is a more potent inducer of NAD^+^ boosting than NR, NMN, or vitamin B3 (NAM and niacin) (14, 17) and we confirmed these observations in macrophages. Both NR and NRH appears to enter the cell through ENTs. However, some differences between these two precursors could explain why NRH is much more potent than NR in boosting NAD^+^ levels. While NR is phosphorylated by NRK into NMN, NRH is phosphorylated by adenosine kinase into NMNH, indicating that these precursors boost intracellular NAD^+^ by different molecular pathways. Another possible difference is the fact that NR is degraded extracellularly by the enzyme CD157/BST1, which is expressed in macrophages. Even though there is no experimental data on NRH degradation by extracellular enzymes, NRH very rapidly enters the cell and can be detected intracellularly (14, 41), suggesting that it may not be degraded extracellularly.

Along with oxidized NAD^+^, NRH also increases NADH and reactive oxygen species (ROS) production, different than other NAD precursors (16). ROS are integral components of multiple cellular pathways, including those regulating immune responses (42). Consequently, NRH may regulate levels of several metabolites that may mediate its cellular effects. However, very little information is available on the effects of NRH supplementation on cellular functions. Work by Hara et al. (43) previously proposed that once cell volume is corrected, NAD is maintained within a tight homeostatic range. However, NRH treatment clearly leads to much greater increases in NAD levels, and may not be subjected to the same homeostatic feedback as other pathways. In some cells, it appears that NRH supplementation may have cytotoxic effects (16). In HepG3 cells, NRH supplementation in a dose-dependent manner altered cell viability, mitochondrial membrane potential, increased mitochondrial superoxide formation, induced mitochondrial DNA damage, and altered mitochondrial respiration. In contrast, the same doses of NRH did not induce cytotoxicity in human embryonic kidney (HEK293T) cells (16). In BMDM supplemented with NRH, we observed an increase in cell viability and no significant long-term changes in cellular respiration in comparison with untreated controls. This suggests that NRH may not have cytotoxic effects under our experimental conditions and, in fact, may improve cellular health. However, we cannot exclude the possibility that the large increase in NAD levels induced by NRH might be inducing cellular stress in macrophages, leading to activation of NF-κB and pro-inflammatory cytokine gene expression.

In Neuro2a and HEK293 cells, NRH has been shown to reverse NAD^+^ depletion caused by genotoxic stress. Cells treated with peroxide or the alkylative genotoxin MMS were depleted of cellular NAD^+^ and underwent cell death. In contrast, cells treated with NRH maintained higher NAD^+^ concentrations and experienced increased cell survival (17). Thus, it appears that NRH can have cell-specific effects on cell survival and toxicity, highlighting the importance of additional studies in various cell types to fully understand the effect of this precursor on cell function and fate. It is also important to differentiate the biology of NAD^+^ from that of NADH. Since NRH can increase both NAD^+^ and NADH, we should investigate in future studies the specific contributions of each metabolite to NRH-mediated functions.

Our results show that in both bone-marrow-derived and THP-1 macrophages, NRH enhanced activation and induced gene expression of several cytokines/chemokines and enzymes expressed by inflammatory M1 macrophages. These results suggest that NAD^+^-boosting agents such as NRH have the potential to be used to reprogram macrophages to a pro-inflammatory phenotype. Elucidating the behavior of macrophages in tumor progression may possibly improve immunotherapy. For example, tumor-associated macrophages (TAMs), have a clear role in supporting tumor progression, by inducing immune suppression through several mechanisms (44, 45). Thus, a potential therapeutic opportunity to increase the macrophage inflammatory phenotype may be to switch TAMs back to an M1 phenotype (45, 46), highlighting the importance of understanding the mechanisms regulating macrophage-mediated inflammation. Additionally, it is important to understand the role of different NAD^+^ precursors in boosting T cell responses, as an alternative way to promote anti-tumor responses (47). In support of this hypothesis, it was recently shown that the CD38-NAD^+^-Sirt1 axis regulates immunotherapeutic anti-tumor T cell responses (48). Moreover, the use of NAD^+^ precursors need to be investigated carefully in the context of inflammatory conditions and diseases, since they have the potential to increase inflammation.

NRH supplementation induced phosphorylation of p65, a hallmark of NFκB activation, indicating activation of this pathway. The NF-κB pathway is one of the main pathways involved in inflammation and cytokine production in macrophages, and pro-inflammatory stimuli such as LPS are potent activators of this pathway (49). Thus, NRH and LPS might activate similar pro-inflammatory pathways in macrophages. At present, we do not know if other NAD precursors may also activate the NF-κB pathway in BMDM. If this is a unique feature of NRH, or it can be achieved with other doses or supplementation times with other precursors, remains to be explored. An important question to be addressed in future studies is how NRH supplementation and NAD^+^ boosting promote activation of the NF-κB pathway. Answering that question will provide valuable information on how NAD metabolism regulates inflammation. In senescent cells, an increase in NAD^+^ was shown to govern the proinflammatory senescence-associated secretome and to correlate with a decrease in AMPK activation, and an increase in p38 and p65 activation (50). Whether a similar mechanism is involved in the regulation of inflammatory cytokine production in macrophages still needs to be investigated.

Another interesting observation from our studies is that IKK, a component of the NF-κB pathway, regulates the gene expression of several enzymes and transporters involved in NAD metabolism, such as *Nmnat1*, *Nmnat3* and *Ent2*. Inhibition of IKK also inhibited the NAD^+^ boosting induced by NRH, possibly because the expression of these key enzymes and transporters was dramatically inhibited. Since little is known about the regulation of the expression of these genes, especially in immune cells, further studies are needed on the interaction between NAD metabolism and the IKK effects mediated by NF-κB-dependent and -independent pathways. These findings will contribute to our understanding of role of NAD metabolism in inflammation and will help us to develop targeted therapies for these pathways.

## Data Availability Statement

The original contributions presented in the study are included in the article/**Supplementary Material**. Further inquiries can be directed to the corresponding author.

## Ethics Statement

The animal study was reviewed and approved by Mayo Clinic IACUC.

## Author Contributions

Conceived the study and participated in its design and coordination: CC and EC. Carried out experiments and data analysis: CC, TP, LS, JZ, SK, GW, and DM. Synthesized NRH: FH and MM. Wrote the manuscript: CC and EC. Reviewed, edited, and/or provided critical scientific revision of the manuscript: TP, SK, GW, MM, AP, and AC-K. All authors contributed to the article and approved the submitted version.

## Funding

This work was supported in part by grants from the Diller Family Foundation, Ted Nash Long Life Foundation, the Glenn Foundation for Medical Research *via* the Paul F. Glenn Laboratories for the Biology of Aging at the Mayo Clinic (EC), the Mayo and Noaber Foundations, National Institutes of Health (NIH) National Institute of Aging (NIA) grants AG-26094 (EC), AG58812 (EC), and CA233790 (EC).

## Conflict of Interest

EC holds a patent on the use of CD38 inhibitors for metabolic diseases that is licensed by Elysium health. EC is a consultant for TeneoBio, Calico, Mitobridge and Cytokinetics. EC is on the advisory board of Eolo Pharma (Santa Fe, Argentina).

The remaining authors declare that the research was conducted in the absence of any commercial or financial relationships that could be construed as a potential conflict of interest.

## Publisher’s Note

All claims expressed in this article are solely those of the authors and do not necessarily represent those of their affiliated organizations, or those of the publisher, the editors and the reviewers. Any product that may be evaluated in this article, or claim that may be made by its manufacturer, is not guaranteed or endorsed by the publisher.

## Supplementary Material

Supplementary Figure 1 NRH is a potent NAD^+^ precursor in immune cells. **(A–C)** Intracellular NAD^+^ levels were measured after supplementation with NAD^+^ precursors (NMN, NAM, NR, and NRH). **(A)** JVM-13 cells were supplemented for 24 hours with 500 μM of NAD^+^ precursors or left untreated (n = 3-4). **(B, C)** Ramos or Jurkat cells were supplemented with 500 μM of NAD^+^ precursors for 18 hours or left untreated (Ctrl) (n = 2-4). **(D)** BMDM treated with 500 μM NRH or 1 ng/ml and 10 ng/ml LPS for 20 hours. Viable cells were counted at the end of the experiment by trypan blue exclusion method (n = 4). **(E)** Representative image of BMDM treated with 500 μM NRH for 20 hours. Cells were visualized and photographed using EVOS XL Imaging System. **(F)** OCR raw data in control and NRH-treated BMDM for 20 hours. Data are mean ± SD. Significance was determined by comparing treatment groups with control untreated samples analyzed by one-way ANOVA with Dunnett’s multiple comparisons. *P < 0.05, **P < 0.01, ***P < 0.001, ****P < 0.0001.

Supplementary Figure 2 Effect of inhibitors of NAD metabolism enzymes on the NRH effect. **(A)** BMDM were treated or not for 1.5 hours with the following inhibitors: 1 μM 5-IT, 10 μM ABT-702, and 10 μM NBTI. Then, cells were left untreated or supplemented with 500 μM NRH for 6 hours. mRNA expression was measured by qPCR and expressed as relative to the control untreated sample (n = 3-4). **(B)** BMDM were supplemented or not for 16 hours with 500 μM NRH. CXCL1 levels in cell culture media were measured by ELISA (n = 4). **(C, D)** BMDM were pretreated for 1.5 hours with the following compounds: 100 μM adenosine, 5 μM Olaparib, 200 nM FK866, and 100 μM APCP. Then, cells were left untreated or supplemented with 500 μM NRH for 6 hours. **(C)** NAD^+^ measurements (n = 3-5). **(D)** mRNA expression measured by qPCR and expressed as relative to control untreated sample (n = 3-4). Data are mean ± SD. Significance was determined by comparing treatment groups with control untreated samples analyzed by one-way ANOVA with Dunnett’s multiple comparisons, or by performing multiple comparisons analyzed by one-way ANOVA with Tukey’s multiple comparisons test (*Il6* in panel **D**). *P < 0.05, **P < 0.01, ***P < 0.001, ****P < 0.0001.
