# Supplementary figures and images for: Dihydronicotinamide Riboside Is a Potent NAD+ Precursor Promoting a Pro-Inflammatory Phenotype in Macrophages
Source: Front Immunol. 2022 Feb 25;13:840246. doi: 10.3389/fimmu.2022.840246 (PMC8913500; doi:10.3389/fimmu.2022.840246)

## Slide 1
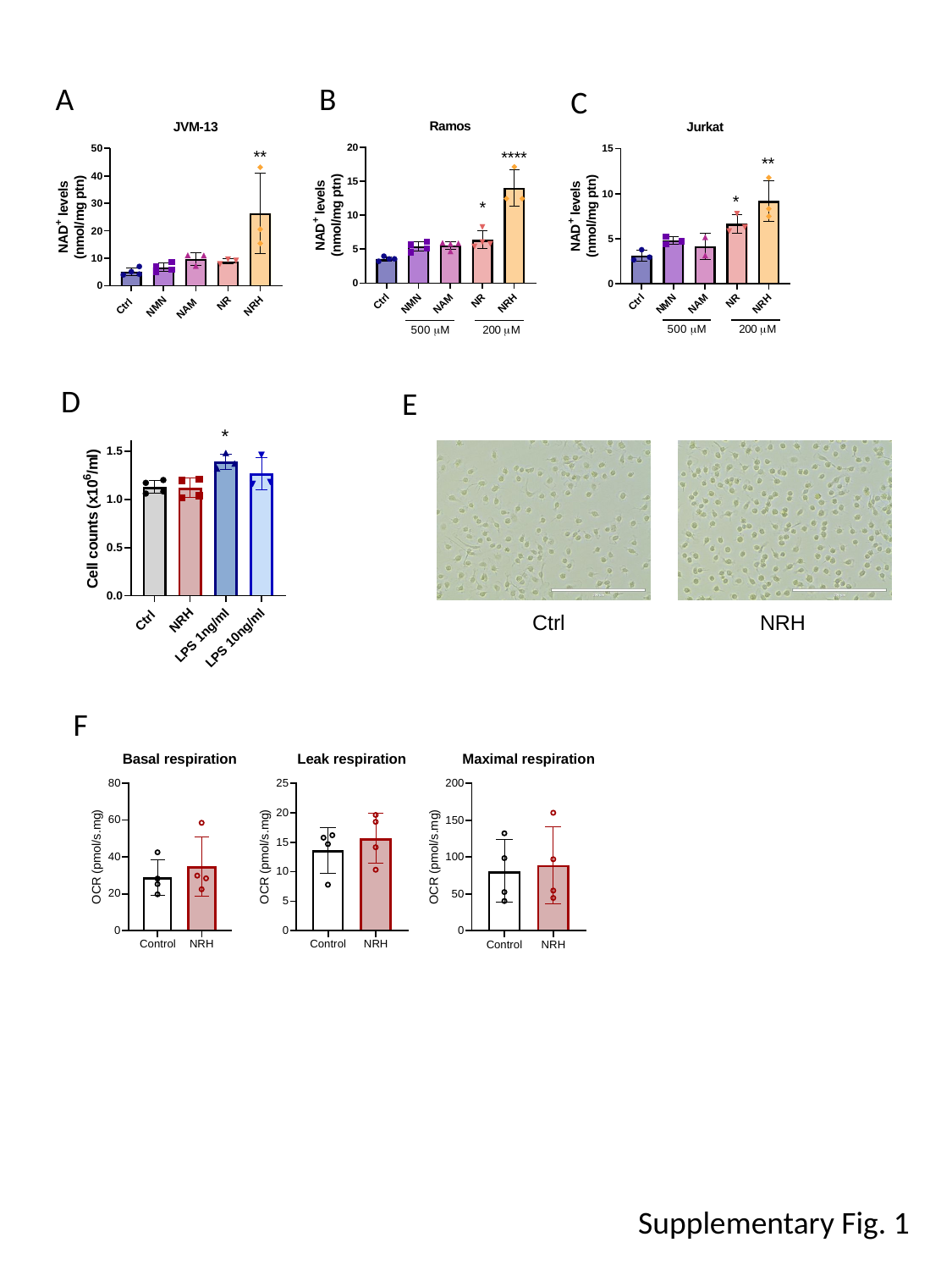

A
B
C
D
E
Ctrl NRH
F
Supplementary Fig. 1

## Slide 2
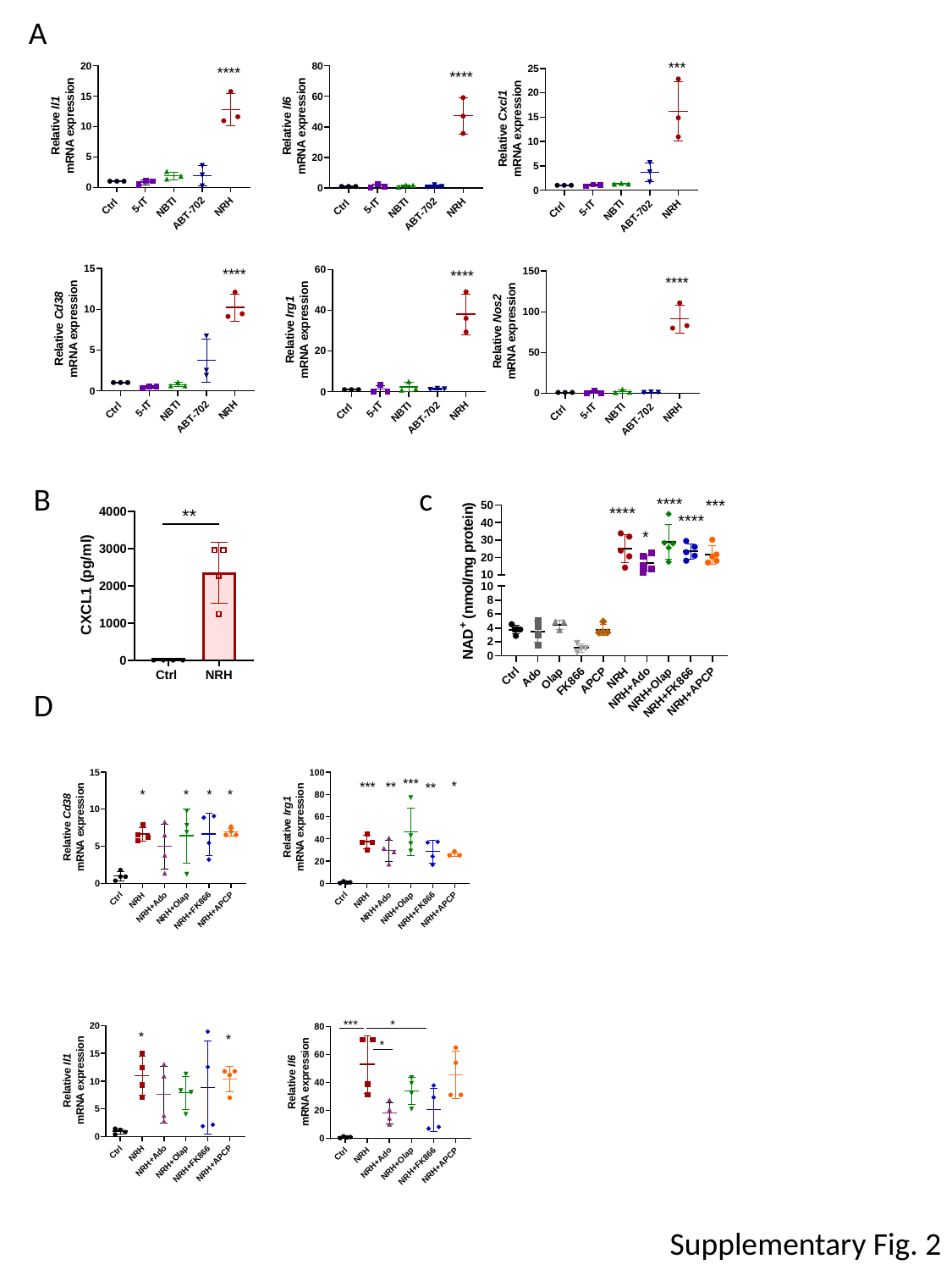

A
c
B
D
Supplementary Fig. 2

Supplement: Supplementary Figure 1 — NRH is a potent NAD+ precursor in immune cells. (A–C) Intracellular NAD+ levels were measured after supplementation with NAD+ precursors (NMN, NAM, NR, and NRH). (A) JVM-13 cells were supplemented for 24 hours with 500 μM of NAD+ precursors or left untreated (n = 3-4). (B, C) Ramos or Jurkat cells were supplemented with 500 μM of NAD+ precursors for 18 hours or left untreated (Ctrl) (n = 2-4). (D) BMDM treated with 500 μM NRH or 1 ng/ml and 10 ng/ml LPS for 20 hours. Viable cells were counted at the end of the experiment by trypan blue exclusion method (n = 4). (E) Representative image of BMDM treated with 500 μM NRH for 20 hours. Cells were visualized and photographed using EVOS XL Imaging System. (F) OCR raw data in control and NRH-treated BMDM for 20 hours. Data are mean ± SD. Significance was determined by comparing treatment groups with control untreated samples analyzed by one-way ANOVA with Dunnett’s multiple comparisons. *P < 0.05, **P < 0.01, ***P < 0.001, ****P < 0.0001. [file Presentation_1.pptx]
